# PKCζ-Mitogen-Activated Protein Kinase Signaling Mediates Crotalphine-Induced Antinociception

**DOI:** 10.3390/toxins13120912

**Published:** 2021-12-20

**Authors:** Bárbara G. de Freitas, Natália G. Hösch, Leandro M. Pereira, Tereza C. Barbosa, Gisele Picolo, Yara Cury, Vanessa O. Zambelli

**Affiliations:** Laboratory of Pain and Signaling, Butantan Institute, São Paulo 05503-900, Brazil; bguimaf@gmail.com (B.G.d.F.); natalia.hosh@esib.butantan.gov.br (N.G.H.); le.marciopereira@yahoo.com.br (L.M.P.); tereza.barbosa@butantan.gov.br (T.C.B.); gisele.picolo@butantan.gov.br (G.P.); yara.cury@esib.butantan.gov.br (Y.C.)

**Keywords:** analgesic peptide, protein kinase C, hyperalgesia, cell-signaling

## Abstract

Crotalphine (CRP) is a structural analogue to a peptide that was first identified in the crude venom from the South American rattlesnake *Crotalus durissus terrificus*. This peptide induces a potent and long-lasting antinociceptive effect that is mediated by the activation of peripheral opioid receptors. The opioid receptor activation regulates a variety of intracellular signaling, including the mitogen-activated protein kinase (MAPK) pathway. Using primary cultures of sensory neurons, it was demonstrated that crotalphine increases the level of activated ERK1/2 and JNK-MAPKs and this increase is dependent on the activation of protein kinase Cζ (PKCζ). However, whether PKCζ-MAPK signaling is critical for crotalphine-induced antinociception is unknown. Here, we biochemically demonstrated that the systemic crotalphine activates ERK1/2 and JNK and decreases the phosphorylation of p38 in the lumbar spinal cord. The in vivo pharmacological inhibition of spinal ERK1/2 and JNK, but not of p38, blocks the antinociceptive effect of crotalphine. Of interest, the administration of a PKCζ pseudosubstrate (PKCζ inhibitor) prevents crotalphine-induced ERK activation in the spinal cord, followed by the abolishment of crotalphine-induced analgesia. Together, our results demonstrate that the PKCζ-ERK signaling pathway is involved in crotalphine-induced analgesia. Our study opens a perspective for the PKCζ-MAPK axis as a target for pain control.

## 1. Introduction

Crotalphine, a structural analogue to an antinociceptive peptide that was first identified in the crude venom from the South American rattlesnake *Crotalus durissus terrificus*, induces a potent and long-lasting antinociceptive effect, mediated by the activation of peripheral opioid receptors [1]. Unlike opioids, treatment with crotalphine for several days does not induce tolerance and withdrawal symptoms. Crotalphine is not an opioid receptor agonist, however, this peptide induces the release of dynorphin A that activates peripheral kappa opioid receptors (KOR) [2]. More recently, attempting to identify the direct molecular targets of crotalphine in pain pathways, it was demonstrated that this peptide partially activates and desensitizes the TRPA1 ion channel at subnanomolar concentrations and this effect is critical for the peptide’s analgesic effect [3].

Mitogen-activated protein kinases (MAPKs) transduce a multiple extracellular stimulus into intracellular effects by modifying the transcription, as well as inducing posttranslational changes in target proteins. There are three major members in the MAPK family: extracellular signal-regulated kinases (ERK) 1/2, C-Jun N-terminal kinase (JNK) and p38, which represent three different signaling pathways [4]. The emerging evidence suggests that MAPKs are involved in nociception and in the development of side effects of drugs, such as morphine. In this regard, peripheral inflammation or nerve injury are followed by spinal activation of ERK1/2, JNK and p38 that seems to increase neuronal excitability and up-regulate transcriptional factors involved in nociception [5]. However, the ERK1/2-signaling activation is essential for morphine and KOR agonists-mediated analgesia in rodents, suggesting that ERK activation may also positively affect the pain outcome [6,7].

It is known that crotalphine increases the level of activated ERK and JNK in cultured sensory neurons from the dorsal root ganglia (DRG) and the ERK levels increase is dependent on the activation of KOR and the protein kinase Cζ (PKCζ) [8]. However, whether the PKCζ-MAPK signaling pathway is involved in crotalphine-induced antinociception has not yet been evaluated in vivo. Considering that crotalphine has a potent and long-lasting analgesic effect in rodents, identifying the intracellular signaling mechanisms responsible for the peptide effects may provide insights to guide the development of better analgesics. Thus, using biochemical and pharmacological strategies, we sought to investigate the role of MAPK in crotalphine-induced antinociception.

## 2. Results

### 2.1. PGE_2_ Is Responsible for the Long-Lasting Antinociceptive Effect Induced by Crotalphine

**Figure 1:**
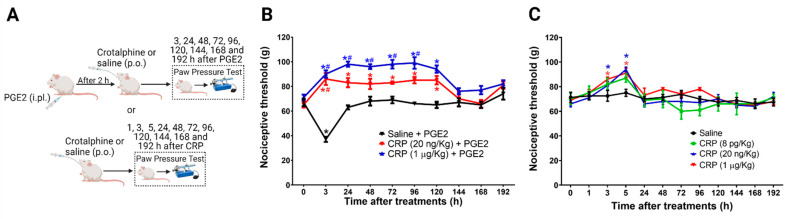
Effect of crotalphine in the mechanical nociceptive threshold of sensitized and non-sensitized rats. (**A**) Schematic representation of treatment and rat paw pressure test. (**B**) PGE_2_ (100 ng/paw) was injected 2 h before the oral crotalphine administration and nociceptive threshold was assessed before (0) and 3, 24, 48, 72, 96, 120, 144, 168 and 198 h after the PGE_2_ injection. Data are presented as mean ± SEM. * significantly different from baseline. # significantly different from the saline + PGE_2_ group, *n* = 6 per group (*p* < 0.05). (**C**) Nociceptive threshold was obtained before (0) and 1, 3, 5, 24, 48, 72, 96, 120, 144, 168 and 198 h after the oral administration of crotalphine. Data are presented as mean ± SEM. * significantly different from the saline group, *n* = 5 per group (*p* < 0.05). The observer was blinded to the experimental conditions.

The data show that the magnitude of the crotalphine antinociceptive effect depends on tissue inflammation [8]. Therefore, we first characterized the effect of one single systemic administration of crotalphine on naïve rats and rats sensitized with an intraplantar injection of Prostaglandin E_2_ (PGE_2_) at day one. The mechanical threshold was assessed 1 h after crotalphine (3 h after PGE_2_) and every day for 8 days (192 h) (Figure 1A). The intraplantar injection of PGE_2_ (100 ng/paw) induced a significant decrease in the mechanical threshold with a peak response occurring 3 h after the administration, compared with the basal values obtained before any treatment, which characterize hypersensitivity. The systemic administration of crotalphine (20 ng/kg or 1 µg/kg, p.o.) blocked the PGE_2_-induced hypersensitivity and increased the nociceptive threshold of the animals when compared with the basal values (analgesia). This effect is detected at 3 h and lasts until 120 h (5 days) after one single administration of crotalphine (Figure 1B). Next, we tested the crotalphine effect in non-sensitized (naïve) rats. A single administration of crotalphine (20 ng/kg and 1 µg/kg, p.o.) induces a significant increase in the mechanical nociceptive threshold (analgesia), starting at 3 h and lasting until 5 h after treatment, when compared with the baseline. A lower dose of crotalphine (8 pg/kg, p.o.) did not change the mechanical threshold (Figure 1C). As expected, no difference in the nociceptive threshold was detected in rats treated with saline (control). Together, these results demonstrate that a single administration of PGE_2_ enhances and prolongs crotalphine-induced analgesia.

### 2.2. Crotalphine Increases the Spinal ERK and JNK and Decreases p38 Activation

**Figure 2:**
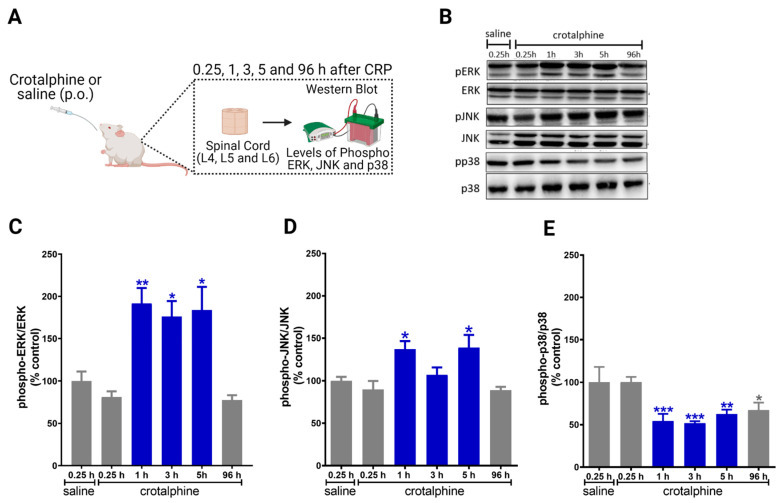
Systemic crotalphine administration increases ERK1/2 and JNK and decreases p38 phosphorylation levels in the spinal cord. (**A**) Schematic representation of treatment and immunoblot. (**B**) Representative blots showing the phosphorylated and total ERK1/2, JNK and p38 levels in the total lysate of spinal cord. Changes in protein expression of ERK 1/2 (**C**), JNK (**D**) and p38 (**E**) MAPKs at different time points were determined by Western blot analysis in lumbar spinal cord extracts from crotalphine or saline-treated rats. Graphs represent the ratio between the phosphorylated protein and the total amount of the targeted protein. Data are presented as mean ± SEM and expressed as % of control (saline) animals. * significantly different from mean values of saline treated animals, *n* = 6 per group (*p* < 0.05). ** significantly different from mean values of saline treated animals, *n* = 6 per group (*p* < 0.01). *** significantly different from mean values of saline treated animals, *n* = 6 per group (*p* < 0.001). The observer was blinded to the experimental conditions.

In order to investigate the potential mechanisms involved in the antinociception induced by systemic crotalphine, we sought to determine whether crotalphine interferes with the expression and phosphorylation of ERK, JNK and p38 (Figure 2A). Importantly, since the inhibition of MEK, an up-stream MAPK kinase, blocks PGE_2_ induced hyperalgesia, the following experiments were conducted in naïve rats, i.e., without sensitization to focus on the mechanism involved in crotalphine-induced analgesia [7]. Moreover, we selected the 1 µg/kg dose of crotalphine for the next experiments.

Crotalphine increases the activation of ERK1/2 in the lumbar spinal cord at 1, 3 and 5 h after administration, when compared with saline-treated rats (Figure 2B,C). Crotalphine also increases the levels of JNK phosphorylation at 1 and 5 h (Figure 2B,D). Conversely, crotalphine decreased the phosphorylation of p38 for up to 96 h after administration (Figure 2B,E). Together, these data indicate that ERK1/2 and JNK are activated in the period in which crotalphine is inducing analgesia, whereas p38 is repressed for a longer period.

### 2.3. Spinal ERK and JNK Activation Participates in Crotalphine-Induced Analgesia

**Figure 3:**
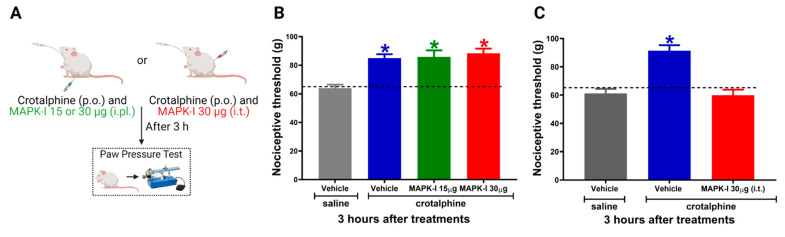
Spinal MAPKs are involved in crotalphine-induced analgesia. (**A**) Schematic representation of treatment and behavior assay. Nociceptive threshold was obtained in the rat paw pressure test before (0) and 3 h after systemic administration of crotalphine (CRP, 1 µg/kg). MEK inhibitor (MAPK-I) was administered immediately after crotalphine (**B**) intraplantarly or (**C**) intrathecally. Data represent mean values ± SEM. * significantly different from baseline (dotted line), *n* = 6 per group (*p* < 0.05). The observer was blinded to the experimental conditions.

To evaluate the functional significance of the increased MAPKs activation in the antinociceptive effect of crotalphine, we used a pharmacological MEK inhibitor (MAPK-I, PD98059) (Figure 3A). First, to investigate whether the peripheral MAPKs participate in crotalphine-induced analgesia, the inhibitor was injected by an intraplantar route in two different doses (15 and 30 µg/paw). MAPK-I did not interfere with crotalphine-induced antinociception (Figure 3B). However, the intrathecal MAPK-I injection (30 µg) completely reversed crotalphine-induced mechanical analgesia, showing that spinal MAPKs, but not intraplantar, are involved in crotalphine’s beneficial effects (Figure 3C). We next examined which MAPK would be responsible for the crotalphine effects.

**Figure 4:**
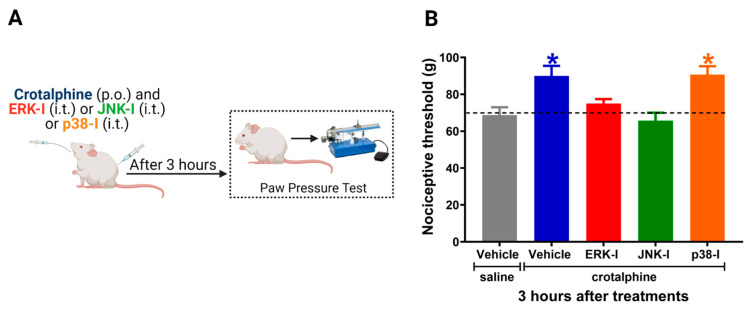
ERK1/2 and JNK inhibitors prevent crotalphine-induced analgesia. (**A**) Schematic representation of the experimental procedure. (**B**) Nociceptive threshold was assessed in the rat paw pressure test before (0) and 3 h after systemic administration of crotalphine (1 µg/kg) with concomitant intrathecal injection of the ERK inhibitor (ERK-I, 30 µg/30 µL), JNK inhibitor (JNK-I, 30 µg/30 µL) or p38 inhibitor (p38-I, 30 µg/30 µL). Data represents mean values ± SEM. * significantly different from baseline (dotted line), *n* = 6 per group (*p* < 0.05). The observer was blinded to the experimental conditions.

The ERK-I and JNK-I intrathecal administration prevented the antinociceptive effect of crotalphine. Nevertheless, the p38 inhibitor did not interfere with crotalphine-induced analgesia (Figure 4B). The control animals injected with the inhibitors alone did not display changes in the nociceptive threshold [7]. Together, these results indicate that crotalphine-induced ERK and JNK activation is responsible, at least in part, for the analgesic effect of this peptide.

### 2.4. Spinal PKCζ Is Involved in the Antinociceptive Effect of Crotalphine

**Figure 5:**
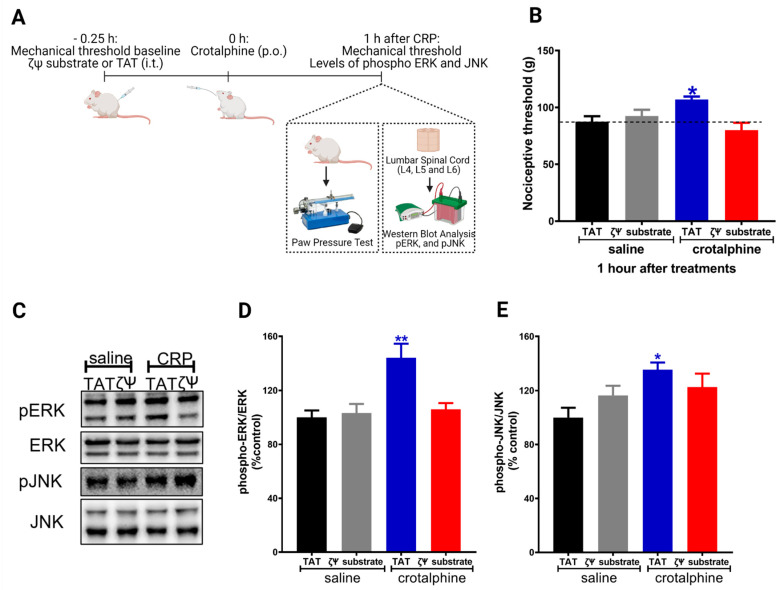
PKCζ mediates crotalphine-induced ERK1/2 activation and antinociception. (**A**) Schematic representation of paw pressure test and experimental procedure. (**B**) PKCζ pseudosubstrate (ζΨ substrate; 3 μg/30 µL) was intrathecally injected 15 min before systemic crotalphine administration (1 µg/kg) and nociceptive threshold was assessed before PKCζ pseudosubstrate injection and 1 h after crotalphine treatment. Data represents mean values ± SEM. *significantly different from baseline (dotted line), *n* = 6 per group (*p* < 0.05). (**C**) Representative blots showing the phosphorylated and total ERK1/2 and JNK levels in the total lysate of the spinal cord. ERK1/2 (**D**) and JNK (**E**) MAPKs were determined by Western blot analysis in lumbar spinal cord extracts from rats treated with crotalphine and the PKCζ pseudosubstrate. Graphs represent the ratio between the phosphorylated protein and the total amount of the targeted protein. Data are presented as mean ± SEM and expressed as % of control (TAT + saline) animals. * significantly different from mean values of TAT + saline treated animals, *n* = 6 per group (*p* < 0.05). ** significantly different from mean values of TAT + saline treated animals, *n* = 6 per group (*p* < 0.01). The observer was blinded to the experimental conditions.

The activation of the κ-opioid receptor increases the phosphorylation of MAPKs (ERK1/2 and JNK) in neuronal and non-neuronal cells [6,9]. In addition, pretreatment of sensory neurons (DRG cells) with a PKCζ pseudosubstrate abolishes crotalphine-mediated ERK1/2 and JNK phosphorylation [8]. Based on this data and on the results showing that spinal ERK1/2 and JNK are involved in crotalphine-induced analgesia, we further investigated whether PKCζ plays a role in crotalphine effects. To examine the PKCζ participation on the mechanical nociceptive threshold, we used the PKCζ pseudosubstrate, which selectively inhibits the atypical PKCζ isozyme [8,10]. The PKCζ pseudosubstrate was injected by an intrathecal route (3 µg) [11] before the crotalphine administration (Figure 5A). As shown in Figure 5B, the analgesia induced by crotalphine was completely abolished by the PKCζ pseudosubstrate.

Finally, to examine the direct role that PKCζ plays in the activation of the MAPKs by crotalphine, we used the PKCζ pseudosubstrate and performed the biochemical studies. Our results show that PKCζ inhibition prevents crotalphine-induced ERK1/2 activation (Figure 5D), without interfering with the JNK (Figure 5E). These data suggest that spinal PKCζ activation mediates crotalphine-induced ERK1/2 activation that culminates in analgesia.

## 3. Discussion

Crotalphine, a 14-amino acid peptide isolated from *C. d. terrificus* venom, has a potent, long-lasting and KOR-mediated antinociceptive effect [1,8,12,13]. In the present study, we showed that a single systemic administration of crotalphine induces analgesia for 5 h in non-sensitized rats. Moreover, when the peptide is administered in PGE_2_ sensitized rats, a potent antinociceptive effect is detected for 5 days. Importantly, the peptide did not induce delayed hypersensitivity, which is a decrease in the nociceptive threshold that follows the analgesic effect, a side effect frequently produced by systemic morphine [7,14]. Together, these results confirm the previous finding showing that a peripheral sensitization enhances the antinociceptive effects of opioid-like drugs [8]. 

Several preclinical studies have shown that opioids activate the MAPK pathway [9,15,16,17] and this activation is usually associated with cellular stress, inflammation and activation of the sensory neuron that ultimately contribute to nociception [18,19]. However, the use of MAPK inhibitors as analgesics in humans is controversial [20] and the clinical trials involve mainly p38 inhibitors [21,22]. Here, we revealed that spinal ERK1/2 and JNK are activated at the same time period that crotalphine induces analgesia. Importantly, the pharmacological disruption of these kinases is sufficient to blunt the antinociceptive effect. Although several studies have shown that the activation of opioid receptors leads to ERK1/2 and JNK activation [23,24], there are few studies correlating the MAPK pathway with analgesia. Recently, Abraham and co-workers (2018) showed that the ERK1/2 signaling is required by KOR agonists to induce analgesia, since the agonist efficacy was reduced in females with estrogen-induced ERK1/2 impairment [6]. Of interest, one systemic dose of morphine in rats promotes analgesia through spinal ERK activation [7], however, whether this effect is mediated by KOR is unknown. Together, these studies suggest that regardless of its up-stream signaling, the same MAPK can activate different signaling pathways simultaneously (essential and detrimental) and therefore affects the effectiveness of MAPK inhibitor as analgesics.

As mentioned before, crotalphine activates ERK1/2 and JNK in cultured sensory neurons mediated by KOR since the selective opioid receptor antagonist, Nor-BNI, prevents this effect [8]. In the present study, we did not check whether KOR is involved in crotalphine-induced ERK1/2 and JNK activation; however, crotalphine induces the release of dynorphin A that activates KOR in vivo [2].

Unlike what was observed for ERK and JNK, the basal levels of spinal p38 phosphorylation were decreased for at least 96 h after crotalphine administration. As expected, the p38 inhibitor did not interfere with the peptide effect. The phosphorylation of p38 is crucial for the activation of the transcription factors that are involved with the synthesis of pro-inflammatory cytokines and neuromediators, such as calcitonin gene-related peptide (CGRP) and substance P [25,26,27]. Aiming to investigate the mechanisms involved in the crotalphine long-lasting effect, we tracked the MAPK levels in the spinal cord for 96 h. Our biochemical data confirm the behavioral results showing that crotalphine may have a prolonged inhibitory effect that lasts up to 96 h. However, there is a limitation in our study since the phosphorylation levels in PGE_2_-sensitized animals was not determined. However, we can speculate from the results obtained with naïve rats that p38 inhibition may contribute to the long-lasting crotalphine-induced analgesia in sensitized rats. Further studies are needed to address this interesting effect.

PKC activation represents an early signaling element in the opioid pathways to ERK and the PKCζ isoform is responsible for mediating KOR-induced ERK phosphorylation [9,15,28]. Additionally, cell culture assays have shown that crotalphine-induced phosphorylation of ERK1/2 and JNK requires PKCζ activation [8]. Thus, to confirm the contribution of PKCζ in crotalphine-induced antinociception, we injected the PKCζ pseudosubstrate intrathecally, which selectively inhibits the atypical PKCζ isozyme, in non-sensitized rats. Our results demonstrate that PKCζ activation is involved in crotalphine-induced analgesia since the PKCζ pseudosubstrate prevented the antinociception. Moreover, Western blot analyses showed that the PKCζ pseudosubstrate decreased the levels of ERK1/2 phosphorylation, but not of JNK, which was increased in the lumbar spinal cord of rats treated with crotalphine. These results are consistent with the previous data showing that crotalphine activates ERK via PKCζ in cultured sensory neurons [8]. The absence of effect on JNK phosphorylation does not exclude that this pathway is modulated by PKCζ, since in sensitized sensory neuron cultures this kinase is inhibited by crotalphine. Moreover, it is important to mention that, given the short half-life of the PKCζ pseudosubstrate, the crotalphine effects were assessed at 1h after treatment.

Additional studies are required to understand which molecules and/or signaling pathways are being modulated by these kinases. However, it is possible that crotalphine and maybe KOR-agonists use PKCζ/ERK1/2 signaling pathway to regulate transcription factors that are essential for the expression of receptors/channels and/or activation of others signaling pathways, which are involved with antinociception [19,23,29,30]. For example, MAPK phosphorylation activates transcription factors, such as cAMP responsive element binding protein (CREB), which regulates dynorphin gene expression [30,31]. 

A number of limitations to our study should be noted; for example, we did not investigate whether crotalphine crosses the blood–brain barrier and directly acts on the spinal cord. We also did not demonstrate whether the effect detected in spinal cord tissue is a consequence of a peripheral action, for example, the peptide acting on sensory fiber synapses with the spinal cord neurons. Further studies are necessary to address these questions.

Taken together, our findings show that crotalphine induces analgesia by down-regulating p38 signaling pathways and activating ERK and JNK in the spinal cord via PKCζ. These results contribute to the understanding of the molecular mechanisms that are involved in the analgesic effect of drugs with opioid activity and opens a perspective for the PKCζ-MAPK axis as a target for pain control.

## 4. Conclusions

In conclusion, our findings demonstrate that a single analgesic dose of crotalphine results in activation of ERK and JNK, and this phenomenon is mediated by PKCζ activation. Targeting the PKCζ-MAPK axis may become an interesting therapeutic alternative to induce analgesia.

## 5. Materials and Methods

### 5.1. Animals

In this study, we used male Wistar rats weighting between 170 and 190 g. The rats were housed in an environment with temperatures of 21 ± 2 °C, and light-controlled with a 12 h/12 h light/dark cycle. Standard food and water were available ad libitum. The procedures were performed according to the guidelines for the ethical use of conscious animals in pain research, following the International Association for the Study of Pain [32]. This study was approved by the Institutional Animal Care Committee of the Butantan Institute (CEUAIB, protocol number 9766020419 (date of approval 19 March 2014) and 1245/14 (date of approval 17 April 2019)). The ARRIVE guidelines was followed while conducting the study.

### 5.2. Chemicals and DrugAadministration

Crotalphine (<E-F-S-P-E-N-C-Q-G-E-S-Q-P-C, where <E is pyroglutamic acid and a disulfide bond between 7C-14C) was synthesized as described by Konno et al. (2008) [1] by the American Peptide Co. (Sunnyvale, CA, USA). The peptide was stored lyophilized at −20 °C until use. For the stock solutions, the peptide was dissolved in sterile distilled water and then diluted in sterile saline immediately before the experiments. Crotalphine at doses of 8 pg/kg, 20 ng/kg or 1µg/kg (oral route) was administered 60 min before the first nociceptive evaluation. PGE_2_ was purchased from Sigma Chemical Co. (St. Louis, MO, USA) and was prepared as previously described [7]. The PGE_2_ was injected (100 ng/paw) in a volume of 100 µL with 0.2% of ethanol [8]. ERK inhibitor (ERK-I), p38 inhibitor (SB20358; p38-I) and JNK inhibitor (SP660125; JNK-I), were dissolved in 5% dimethyl sulfoxide (DMSO) and administered intrathecally (i.t.) at the dose of 30 µg. PD98059, a MEK inhibitor, was dissolved in 5% DMSO and administered intraplantarly (i.pl.) (10 or 30 µg/paw) or intrathecally (30 µg) [33]. The peptide ζ-pseudosubstrate ([C]SIYRRGARRWRKLYRAN; amino acids 105-121 in ζ-PKC) was synthesized by Zhejiang Ontores Biotechnologies Co. (Hangzhou, Zhejiang, China) and fused to the cell permeable TAT peptide (YGRKKRRQRRR) transduction domain peptide. The control group received the TAT peptide (American Peptide Co., Sunnyvale, CA, USA). Stock solutions were made in sterile distilled water and diluted in sterile saline. This peptide was administered intrathecally at the dose of 3 µg. In summary, for these intrathecal injections, the animals were anesthetized with 2% isoflurane and the needle was placed in the subarachnoid space on the midline between L4 and L5 vertebrae [34], with a maximum volume of 50 µL. The animals recovered consciousness approximately 1 min after discontinuing the anesthetic.

### 5.3. Behavioral Assessment

#### Evaluation of the Antinociceptive Effect

The behavioral tests were performed between 9:00 am and 4:00 pm. To assess the mechanical nociceptive threshold, we used the rat paw pressure test [35] (Ugo Basile, VA, Italy). The pressure was recorded before and for up to 192 h after the crotalphine treatment or PGE_2_ injection. Testing was conducted blind to the group designation. To reduce stress, the rats were habituated to the testing procedure a day before experimentation. In this test, an increasing amount of force (measured in g) was applied to the hind paw of the rat and interrupted when the animal withdrew the paw. The force necessary to induce this reaction was recorded as the mechanical nociceptive threshold. A maximum pressure of 250 g (i.e., cut-off) was established to avoid damage to the paw.

### 5.4. Biochemical Studies

#### Western Blot Analysis

Spinal cord tissues (L4–L6) were collected and homogenized in a lysis buffer containing a protease and phosphatase inhibitor cocktail (Sigma-Aldrich). Protein concentrations of the samples were determined using a Bradford assay [36]. Total protein of 30 μg was separated on SDS-PAGE gel (10% gradient gel) and transferred to nitrocellulose membranes (BioRad, Santo Amaro, SP, Brazil). The membranes were blocked for 120 min with 5% bovine serum albumin (BSA) or non-fatty milk and incubated overnight at 4 °C with a primary antibody against phospho-ERK, phospho-JNK, phospho-p38 or non-phosphorylated forms of these proteins (1:1000; Cell Signaling Technology, Danvers, MA, USA). The membranes were then incubated in the correspondent peroxidase-conjugated secondary antibody (1:5000; antirabbit or antimouse, Sigma-Aldrich, St. Louis, MO, USA, cat. numbers A0545 and A8919, respectively) for 120 min at room temperature. The enhanced chemiluminescence method was used to develop the filters (Amersham GE Healthcare Bio-Sciences Corp.; Piscataway, NJ, USA). The densitometric data were analyzed using the UVITEC Cambridge software (UVITEC Cambridge, Cambridge, UK) and normalized to the total protein (not phosphorylated).

### 5.5. Statistical Analyses

Statistical analysis was performed using GraphPad Prism 8 program (GraphPad Software Inc., San Diego, CA, USA). The results are presented as the mean ± SEM. The statistical evaluation of the data was performed using two-way analysis of variance (ANOVA) with post hoc testing by Tukey for Figure 1, Figure 3, Figure 4 and Figure 5B. Figure 2 and Figure 5D,E were analyzed using One-way ANOVA followed by Tukey’s post hoc test. A value of *p* < 0.05 was considered significant.
